# Photothermally responsive icariin and carbon nanofiber modified hydrogels for the treatment of periodontitis

**DOI:** 10.3389/fbioe.2023.1207011

**Published:** 2023-05-16

**Authors:** Xiangjiao Zheng, Zhiqiang Dong, Zepeng Liang, Yu Liu, Xiaowei Yin, Mofei Han, Zhongcheng Cui, Xifan Mei, Xiuqiu Gao

**Affiliations:** Jinzhou Medical University, Jinzhou, China

**Keywords:** ICA, CNF (carbon nanofiber), near infared, periodontitis, hydrogel

## Abstract

**Introduction:** Periodontitis is a chronic inflammatory disease brought on by various bacteria, and effective antibacterial, anti-inflammatory and alveolar bone regeneration are the main goals of treating periodontal disease.

**Methods:** In the current work, we employed Icariin (ICA) into a hydrogel modified with carbon nanofiber (CNF) to create a multifunctional composite nanoplatform. The composite was activated in the near infrared (NIR) to treat periodontitis.

**Results:** The antibacterial results showed that the ICA+CNF@H showed 94.2% and 91.7% clearance of *S. aureus* and *E. coli*, respectively, under NIR irradiation. *In vitro* experiments showed that NIR-irradiated composites suppressed inflammatory factor (IL-6) and ROS expression and up-regulated the performance of anti-inflammatory factor (IL-10) in RAW264.7 cells. At the same time, the composites promoted the production of osteogenic factors in BMSCs, with an approximately 3-fold increase in alkaline phosphatase activity after 7 days and an approximately 2-fold increase in the rate of extracellular matrix mineralization after 21 days. *In vivo* tests showed that the alveolar bone height was clearly greater in the ICA+CNF@H (NIR) group compared to the periodontitis group.

**Discussion:** In conclusion, ICA+CNF@H under NIR irradiation achieved a synergistic effect of antibacterial, anti-inflammatory, reduction of reactive oxygen species and promotion of osteogenesis, offering a novel approach for treating periodontitis.

## 1 Introduction

Periodontal disease is currently the leading reason for adult tooth loss, and periodontitis is considered to be one of the commonest oral diseases in the world (Slots, 2017). Periodontitis refers to a chronic and damaging inflammatory disorder of the periodontal supporting tissues (gums, periodontium and alveolar bone) caused by microbial infection (Konkel et al., 2019). As the disease progresses, it causes destruction and absorption of the alveolar bone as well as loss of periodontal attachments, eventually leading to loosening, ectasia and loss of teeth (Kwon et al., 2021). Research indicates that periodontitis is linked to systemic health and can increase exposure to numerous diseases, like atherosclerosis, adverse effects of pregnancy, rheumatoid arthritis, diabetes, aspiration pneumonia, and cancer (Michaud et al., 2017; Wen et al., 2019; Figuero et al., 2020; Sanz et al., 2020; Wu et al., 2020; Imai et al., 2021). Treatment of periodontitis is not only about controlling inflammation, but more importantly, preventing periodontal tissue destruction, inducing alveolar bone regeneration and restoring the function of a healthy oral system. The current clinical treatment of periodontitis is mainly through basic treatment and surgery (Van der Weijden and Timmerman, 2002). Although these treatment modalities have been effective in treating periodontitis, they often separate antibacterial and bone regeneration, while facing problems such as drug resistance, trauma, and unclear effects. Therefore, the preparation of multifunctional nanomaterials with antibacterial, anti-inflammatory and bone regeneration-promoting functions is an active and effective approach to treating periodontitis.

**SCHEME 1:**
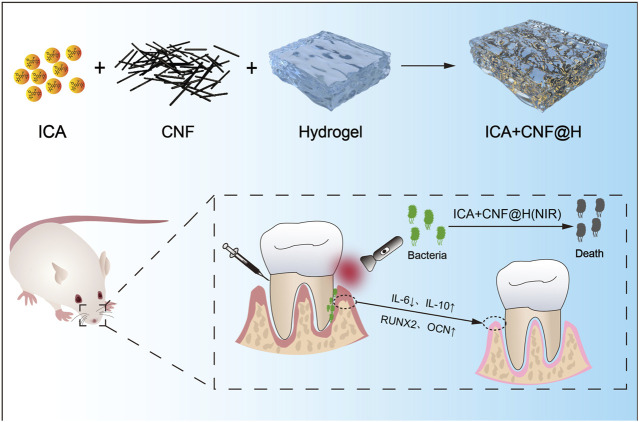
Synthetic ICA + CNF@H. The composite is activated in the near infrared and combines antibacterial, anti-inflammatory and osteogenic activity to treat periodontitis.

With the rapid development of biomaterials, multifunctional composite nanoplatforms triggered by near-infrared light for local and precisely targeted therapies by physical means have attracted interest (Wu et al., 2021; Xu et al., 2022). Nowadays, the commonest NIR light treatment strategies are PTT, PDT and drug delivery systems (Ying-Yan et al., 2021; Chelushkin et al., 2022; Lin et al., 2022; Pang et al., 2023). PTT uses a light absorbing device to transform light energy directly to heat energy and ablate the surrounding abnormal tissue by local heating to achieve the treatment effect (Zhi et al., 2020; Dong et al., 2023). PDT, on the other hand, is a photosensitizer that produces reactive oxygen species under the irradiation of NIR, which has a specific phototherapeutic impact on diseased structures (Donohoe et al., 2019). NIR light possesses excellent penetrating power in tissues, and studies have shown that a wavelength of 810 nm can penetrate 51% of the skull, while that of 660 nm can only reach the surface of cortical bone (Salehpour et al., 2019). At the same time, NIR light is easily controlled in time and space, and as a physical external stimulus, NIR light has a little toxic or destructive effect on normal tissue (Hu et al., 2019a). CNF are fibrous carbon nanomaterials made from multiple layers of graphite sheets curled together (Song and Shen, 2014). It is a carbon material between carbon nanotubes and ordinary carbon fibers, with superior thermal conductivity, mechanical properties, low cytotoxicity, good bioactivity and chemical stability, and is now widely used in the field of biomaterials (Stout, 2015). Hydrogels are a new type of solid-like viscoelastic novel drug delivery system, their hydrophilic nature allows them to absorb large amounts of water. Hydrogels can encapsulate a variety of therapeutic drugs and release them in a variety of ways to treat disease, making them a suitable choice for drug delivery in the oral environment (Hosseinpour-Moghadam et al., 2021). ICA is a major active constituent of Icariophyceae, a genus of plants in the Berberaceae family, with a variety of pharmacological and biological activities, such as promotion of bone regeneration and bone repair, estrogenic effects, anti-inflammatory and antioxidant effects, neuroprotective effects, anti-atherosclerotic effects and anti-tumour effects (Wu et al., 2012; Fang and Zhang, 2017; Wang et al., 2018; Sun et al., 2019; Xu et al., 2020; Wang et al., 2021; Fu et al., 2022). Bone marrow mesenchymal stem cells (BMSCs) are multifunctional stem cells located in the bone marrow stroma and have the potential to produce various cell lines including osteoblasts, chondrocytes and adipocytes (Chu et al., 2020). The development of ICA as an adjuvant periodontal drug has great potential (Zhu et al., 2022). Therefore, we prepared ICA + CNF@H, which were effective antibacterial under NIR light irradiation. And the composites reduced the exposure of inflammatory factors (IL-6) and reactive oxygen species (ROS) in RAW264.7 cells, altering the resorption of alveolar bone due to the periodontal inflammatory environment. Meanwhile, ICA + CNF@H regulated the expression of osteogenic factors in BMSCs and promoted bone tissue regeneration, providing a novel option to effectively treat periodontitis. The detailed study protocol of iICA+CNF@H(NIR) in the treatment of periodontitis is shown in Scheme 1.

## 2 Materials and methods

### 2.1 Experimental reagents and materials

Alpha-modified Eagle’s Medium (α-MEM), penicillin-streptomycin (PS), 10% foetal bovine serum (FBS) and 0.25% trypsin-ethylenediaminetetraacetic acid (EDTA) were purchased from Gibco Company (United States). 4% paraformaldehyde fixative (4% PFA) and 2-(4-aminophenyl) - 6-indolyl methylamino dihydro ether (DAPI) were purchased from Shanghai BTS Biotechnology Co. Ltd. Calcein AM/PI Cytotoxicity Assay Kit was purchased from Solarbio (China). *Staphylococcus aureus* (*Staphylococcus aureus*) and *E. coli* (*Escherichia coli*) required for the present study were purchased through the American Type Culture Collection (ATCC) purchased from the Liaoning Provincial Center for Disease Control and Prevention. ICA, PVA (polyvinyl alcohol) and Alg (sodium alginate) were obtained from Aladdin Reagents Ltd. (China).

### 2.2 Preparation of material

First, 4 mg of CNF was incorporated into 20 mL of Milli-Q water and ultra-sonicated over 1 h. Subsequently, 2 mL (1 mg/mL) of ICA solution was added and stirred for 12 h under fast stirring conditions (1,000 r/min) to allow sufficient adsorption. The precipitates were gathered using a filtration unit, cleaned a few times, and the gathered precipitates were freeze-dried to yield ICA + CNF particles.

The following procedure was performed to produce hydrogels. Add 0.1 g of Alg to 10 mL of 8% PVA solvent in a constant temperature bath at 50°C and mix thoroughly with a magnetic stirrer at 350 r/min until homogeneous for 30 min. Finally, 1 mg of ICA + CNF powder was incorporated into the hydrogel liquid and thoroughly blended under magnet agitation to obtain the composite of ICA + CNF@H.

### 2.3 Characterization

The morphological characteristics of the CNF were achieved via scanning electron microscopy (SEM, HitachiSU8020, Tokyo, Japan). The composition of the sample was recognized via Fourier transform infrared spectroscopy (FTIR, Shimadzu, Kyoto, Japan). The protein of interest expression in the cells was photographed employing a confocal laser scanning microscope (CLSM, Leica TSCSP5 confocal unit). UV–vis spectrophotometer (PerkinElmer Lambda 605S UV–vis spectrometer). Expression of the relevant proteins in cells was photographed with a CLSM (Leica TSCSP5 confocal unit).

### 2.4 Rat BMSCs cell culture

Eight-week-old clean-grade Sprague Dawley (SD) rats were obtained from the Experimental Animal Center of Jinzhou Medical University. Rats were euthanized and disinfected using 75% alcohol for 5 min. The intact tibia and femur were removed and placed into sterile PBS, tissues such as muscle and fascia were removed, and the articular portions of the ends of the long bones were cut off. Using a 5 mL syringe, the bone marrow cavity is repeatedly rinsed with an intact cell culture containing 20% fetal bovine serum until the surface is white. The collected bone marrow suspension was reinserted into a new sterile culture dish and the fluid was replaced in every 3 days, and the cells were passaged when they reached approximately 90% growth. Cells used in subsequent experiments were of the 3rd to fifth generation (Jin et al., 2018).

### 2.5 Cell biocompatibility

The effect of ICA + CNF@H on the cell vitality was determined by CCK-8 method. BMSCs cells were inoculated in 96-well plates at a concentration of 5,000 cells/well with a medium volume of 100 μL and cultured at 37°C and 5% CO_2_ for 24 h. Cells were processed for 1, 3 and 7 days with blank control and different materials, respectively. 10 μL of CCK-8 solution was added to each hole, the incubation was carried out for 2 h, and the absorbance was measured at 450 nm using an enzyme marker.

The biosafety of ICA + CNF@H was tested by live-dead staining of cells. ICA + CNF@H and ICA + CNF@H (NIR) were co-cultured with BMSCs for 1, 3 and 5 days, and the effect of composites on BMSCs was examined employing the Calcein AM/PI Cytotoxicity Assay Kit (Solarbio, China). The red fluorescent nucleic acid dye PI is able to penetrate impaired cell walls for labeling dead cells. In addition, Calcein green gets into living cells and is broken down to Calcein by intracellular esterase cleavage, emits intense green fluorescence and stays inside the living cells. Cells were incubated in the staining solution for 30 min and photographed under a fluorescent microscope for observation.

### 2.6 Fluorescence detection of ROS

The effect of ICA + CNF@H on RAW264.7 intracellular ROS was observed via the ROS Assay Kit (Beyotime). RAW264.7 cells were incubated for 24 h, then the medium was removed and fresh medium including LPS, ICA + CNF@H, and ICA + CNF@H(NIR) was added for 5 h, respectively. After irradiating the last group for 10 min using an 808 nm laser, the intracellular ROS were measured using a ROS Assay Kit. DCFH itself does not fluoresce and can be oxidized to the fluorescent product DCF. Fluorescence imaging was recorded immediately after 30 min of DCFH-DA incubation to observe the fluorescence intensity of cellular ROS.

### 2.7 Osteogenesis induction

The effect of composites on BMSCs when differentiating toward osteogenesis was detected by osteogenic induction. BMSCs were spread in six-well plates, 5×10^4^ cells were added to each well, complete medium was added and replaced with osteogenic induction solution after 24 h (50 μM ascorbic acid, 10 mM β-phosphoglycerol, 100 nM dexamethasone, 10% FBS and α-MEM medium). Cells were grown by osteogenic differentiation medium containing different materials, and the medium was changed at 3-day intervals. Alkaline phosphatase was stained on day 7. Cells were removed, fixed in 4% paraformaldehyde for 30 min and subjected to staining with an alkaline phosphatase kit. Following dyeing, cells were cleaned with PBS and see on microscope and camera. ALP activities were also determined with an alkaline phosphatase assay kit. Alizarin Red S staining was carried out after 21 days induction. Cells were immobilized in 4% (w/v) paraformaldehyde for 30 min and washed 3 times with PBS. The wells were processed using 1 mL of freshly prepared 3% (w/v) Alizarin Red S solution (Sigma-Aldrich, Missouri, United States) per well and cultured in darkness for 30 min. Calcium nodules were analyzed qualitatively using a digital camera and microscope. To perform quantitative measurements, three absorption readings were measured at 562 nm for each group after 20 min of separation with 10% (v/v) cetyl pyridinium chloride monohydrate (Sigma-Aldrich).

### 2.8 Cellular immunofluorescence assay

ICA + CNF@H and ICA + CNF@H(NIR) were incubated with BMSCs or RAW264.7 (1 × 10^5^ cells/mL) in confocal glass culture dishes for 24 h. The cells have been rinsed 3 times with PBS for 5 min each. Cells were immobilized in 4% PFA for 40 min. After treatment with 0.1% Triton X-100 (PBS) for 20 min at ambient conditions, normal goat serum was added and cells were closed for 2 h at ambient condition. The corresponding antibodies prepared (IL-6, IL-10, RUNX2, OCN) were added and cultured in the cells for 12 h at 4°C in a refrigerator. On the next day, the primary antibody was recovered, and the secondary antibody was incorporated into goat serum and hatched with the cells for 2 h at ambient condition. Cells were rinsed with PBS and stained with DAPI for 15 min. Finally, images of cells were captured by confocal microscopy.

### 2.9 Periodontitis model

A rat periodontitis model was established using male SD rats (200–230 g) to test the therapeutic effect of ICA + CNF@H(NIR) on periodontitis assessment. The rats were anesthetized by intraperitoneal infusion with 1.5% sodium pentobarbital (35 mg/kg), and the rats were ligated with 0.2 mm diameter orthodontic ligature and silk threads on both maxillary first molars, tied on the palatal side, and the ligature wire was put in the gum sulcus as much as it could, and the normal rats were fed food and pure water from the day of the experiment. Twenty-four rats were selected at random into blank group, periodontal group, ICA + CNF@H group and ICA + CNF@H (NIR) group. The hydrogel treatment was started on the second day after modeling (10 min once every 2 days) at each treatment to check whether the ligature wire was dislodged, to observe the rats’ diet and changes in gait, behavior and other activities, and to check the clinical manifestations of periodontal tissues. On the 30th day, all SD rats were anesthetized, executed and taken, and the therapeutic effect of composites on rats with periodontitis was evaluated by observing the distance of ABC-CEJ of the first molar of the rats through the body view microscope. All SD rats were supplied from the SPF-class laboratory animal center of Jinzhou Medical University and kept in accordance to the standard of alternating light and dark every 12 h. All experimental rats met the first-class animal standard of the Ministry of Health.

### 2.10 Histopathological examination

Specimens of rat maxillary alveolar bone were taken, fixed with 4% paraformaldehyde for 24 h, decalcified by 17% EDTA for 4 weeks, specimens were dehydrated in gradient concentration ethanol solution, transparently treated with xylene, and embedded with paraffin wax to get paraffin sections of 5 μm thickness. The sections were colored by HE Kit and Masson Kit, dehydrated transparently with ethanol and xylene, sealed with neutral adhesive, and placed under the microscope to observe the morphological manifestations of periodontal histology in each group of rats.

### 2.11 *In vitro* antimicrobial properties of the materials

The antibacterial performance of ICA + CNF@H was examined by bacterial agar plate method. All equipment and samples were exposed to UV light for 1 h before the experiment. *S. aureus* or *E. coli* were placed into Luria-Bertani (LB) medium at 37°C, respectively, and incubated overnight at a constant temperature of 37°C until the bacteria reached the logarithmic growth phase for the next experiment. The experiment was classified as Control (blank group), ICA + CNF@H group, and ICA + CNF@H (NIR). One mL of bacterial stock solution was diluted in gradient to 2 × 10^8^ CFU/mL^-1^, respectively, and the composites were co-incubated with *S. aureus* or *E. coli*, and the light group was irradiated under NIR light (irradiation time and frequency of the light group). 100 μL of bacterial suspension was taken and uniformly laid down on agar plates, and the bacterial communities were counted after 24 h of incubation.

### 2.12 Bacterial biofilm formation experiments

The ability of the composite to resist bacterial plaque biofilm was assessed using the *S. aureus* biofilm model. Inoculate 2 mL of *S. aureus* (2 × 10^8^ CFU/mL^-1^) in a 24-well plate and incubate in a 37°C incubator for 48 h. After it was determined that the bacterial plaque biofilm was successfully established, several chemicals were added to the bacterial plaque biofilm and culture was further carried out for 24 h. Afterward, methanol was fixed and stained with crystalline violet (CV), with darker colors representing more bacterial plaque biofilm, and the bacterial plaque biofilm was recorded by microscopy The amount of biofilm was recorded by microscopy. Biofilms were rinsed with sterile deionized water to eliminate unbound dye. Bound CVs were solubilized with anhydrous ethanol and absorption at 590 nm was calculated for all samples using an enzyme marker.

### 2.13 Statistical analysis

Each of the independent studies was repeated three times under appropriate conditions. All charts were created with GraphPad Prism software, version 6. A one-way analysis of variance (ANOVA) was applied to the analysis of statistical significances using *post hoc* Tukey’s multiplicative comparisons check. Differences among the groups were found to be statistically valid at *p < 0.05, **p < 0.01, ***p < 0.001.

## 3 Results

### 3.1 Material properties and characteristics

**FIGURE 1:**
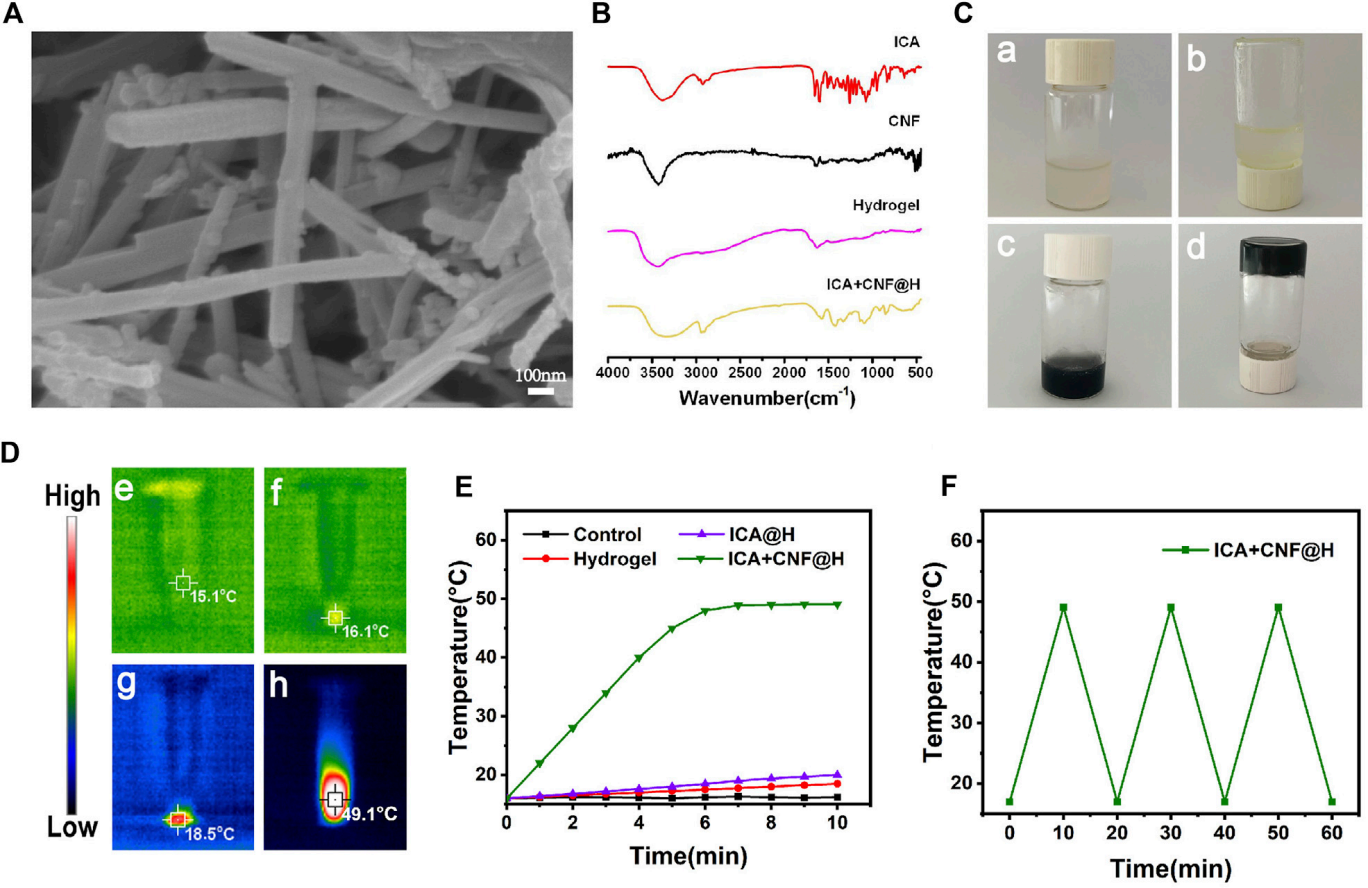
Material Characterization. **(A)** SEM images. **(B)** FTIR spectra of samples. **(C) **Digital photos of ICA + CNF@H. **(D)** Infrared thermogram of the samples following 10 min of radiation exposure. **(E)** Temperature variation profiles of various kinds of materials following 10 min of 808 nm NIR irradiation. **(F)** Photothermal conversion stability of ICA + CNF@H.

The synthesized products were described through SEM and FTIR spectroscopy. The morphology and structure of the CNF were observed by SEM, which clearly showed homogeneous nanofibrous features of CNF with a diameter of approximately 75 nm (Figure 1A). The successful preparation of the composite hydrogel ICA + CNF@H could then be confirmed by FTIR spectroscopy and the chemical stability of ICA in ICA + CNF@H was confirmed using FTIR spectroscopy data (Figure 1B). ICA + CNF@H showed a characteristic peak of ICA at 2,924 cm^-1^, indicating that ICA is well maintained biologically active in the composite. We also observed the mobility of ICA + CNF@H and pure hydrogels. Plots a and b as in Figure 1A show the good mobility of the pure hydrogel, while c and d show the gelation phenomenon after the addition of ICA + CNF to the hydrogel, indicating that ICA + CNF@H has good gelation properties. The photothermal properties of ICA + CNF@H were evaluated by irradiating different sample solutions with a NIR laser to obtain thermographic images and heating curves (Figures 1D, E). After 10 min of continuous laser irradiation, the solution temperatures of all units were 15.1°C (PBS), 16.1°C (Hydrogel), 18.5°C (ICA@H) and 49.1°C (ICA + CNF@H), respectively. ICA + CNF@H exhibited good photothermal performance, respectively. Due to the good photothermal properties of CNF irradiation in the NIR light could rapidly warm up to a maximum temperature of 49.1°C. In the next cycle, when irradiation was stopped, cooling to room temperature showed excellent photothermal stability (Figure 1F). We examined the UV absorption spectra of ICA + CNF@H at different time points to detect the release of ICA at different time periods (Supplementary Figure S1). A longer drug release is more beneficial for the treatment of periodontitis. For better manoeuvrability of oral treatment, we prepared the composite as an injectable type (Supplementary Figure S2). We could push the material out of the syringe smoothly without greater resistance, indicating that ICA + CNF@H has better injectability.

### 3.2 *In vitro* antimicrobial properties of materials

**FIGURE 2:**
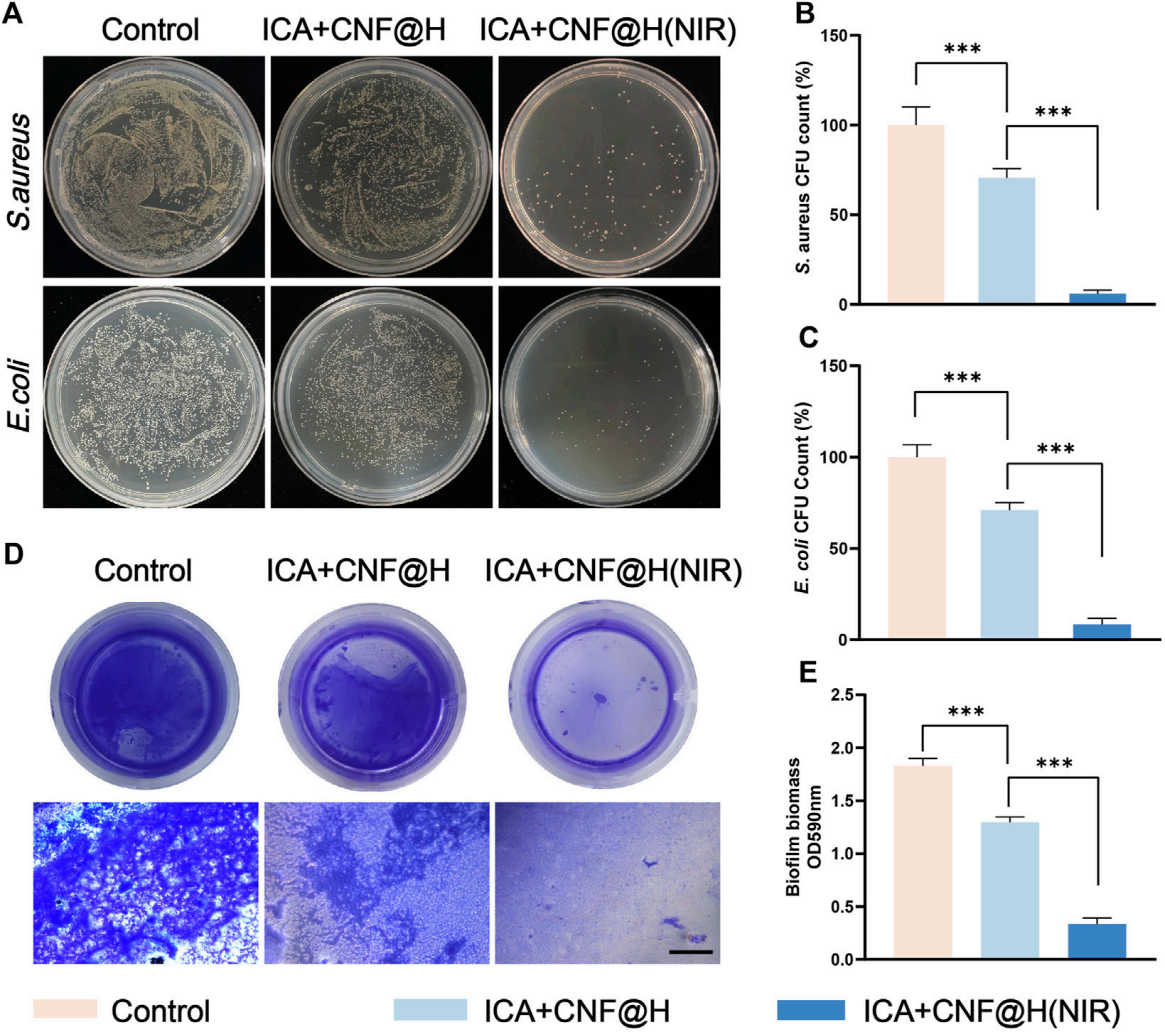
Antibacterial properties of the materials. **(A)** Photos of agar plates of various treated groups. **(B, C)** The respective CFU numbers of *S. aureus* and **(E)** *coli* under different types of processing. **(D)** Photographs of crystalline violet-stained bacterial biofilms and microscopic images; scale bar = 500 μm. **(E)** The absorbance of crystalline violet staining at 590 nm of *S. aureus* biofilms after different groups of treatments. *p < 0.05, **p < 0.01, ***p < 0.001.

The prevention and treatment of periodontitis focus on the pathogenic effect of removing bacteria that are present in the periodontal pockets (Hu et al., 2019b). In clinical practice, periodontitis can be effectively treated by scraping away the bacterial deposits under the gums, but mechanical cleaning cannot completely eliminate the bacteria in the periodontal pockets and the application of antibacterial medication is also important (Mombelli, 2018). The progression of periodontitis is strongly associated with the presence of bacteria in the periodontal micro-Environment. The long-term persistent presence of bacteria can damage the periodontal microenvironment. *S. aureus* and *E. coli* are 2 commonly found drug-resistant species of bacteria in the oral environment. We measured the antibacterial capacity of various materials towards two distinct strains of bacteria by incubating bacteria treated with different materials by agar diffusion method. The results showed that the controls showed a significant amount of colonies on the agar plates, while the ICA + CNF@H reduced the amount of bacterial colonies in comparison with the controls and showed some inhibition of *S. aureus* and *E. coli*. However, the ICA + CNF@H (NIR) group had the lowest bacterial colony counts and showed excellent antibacterial performance (Figure 2A). Quantitative evaluation of colonies of *S. aureus* and *E. coli* showed 94.2% and 91.7% inhibition, respectively (Figures 2B, C), indicating that the excellent photothermal capacity of ICA + CNF@H can inhibit bacteria successfully.

Previous literature has confirmed that the recurrence of periodontitis is closely relate to the generation of bacterial biofilms, which keep the periodontal environment in an inflammatory state, and that prolonged inflammation can destroy the alveolar bone and cause unacceptable damage (Herrera et al., 2008; Seneviratne et al., 2011). Bacteria in biofilms are harder to eradicate compared to planktonic bacteria, so eliminating bacterial biofilms is critical to antimicrobial performance (Lamont et al., 2018). To further test the antibacterial ability of ICA + CNF@H(NIR) against bacterial plaque biofilms, we treated biofilms formed by *S. aureus* with composite materials. The bacterial biofilm was stained by crystalline violet, and more dark bluish color and more biofilm stained by crystalline violet indicated poor antibacterial performance. The experimental results showed that the blank control group showed the darkest blue color (Figure 2D) and the highest absorbance (Figure 2E). The solution of ICA + CNF@H(NIR) was significantly lighter in color (Figure 2D) and lower in absorbance (Figure 2E) compared to the control. This indicates that the irradiation of the composites in the NIR stimulated the photothermal properties of the composites, thus exhibiting better antibacterial properties, and is consistent with the experimental results of the agar diffusion method. Based on these data it was demonstrated that ICA + CNF@(NIR) had the best antimicrobial performance, offering a new strategy for the antimicrobial aspect of periodontal treatment.

### 3.3 Effect of materials on BMSCs

**FIGURE 3:**
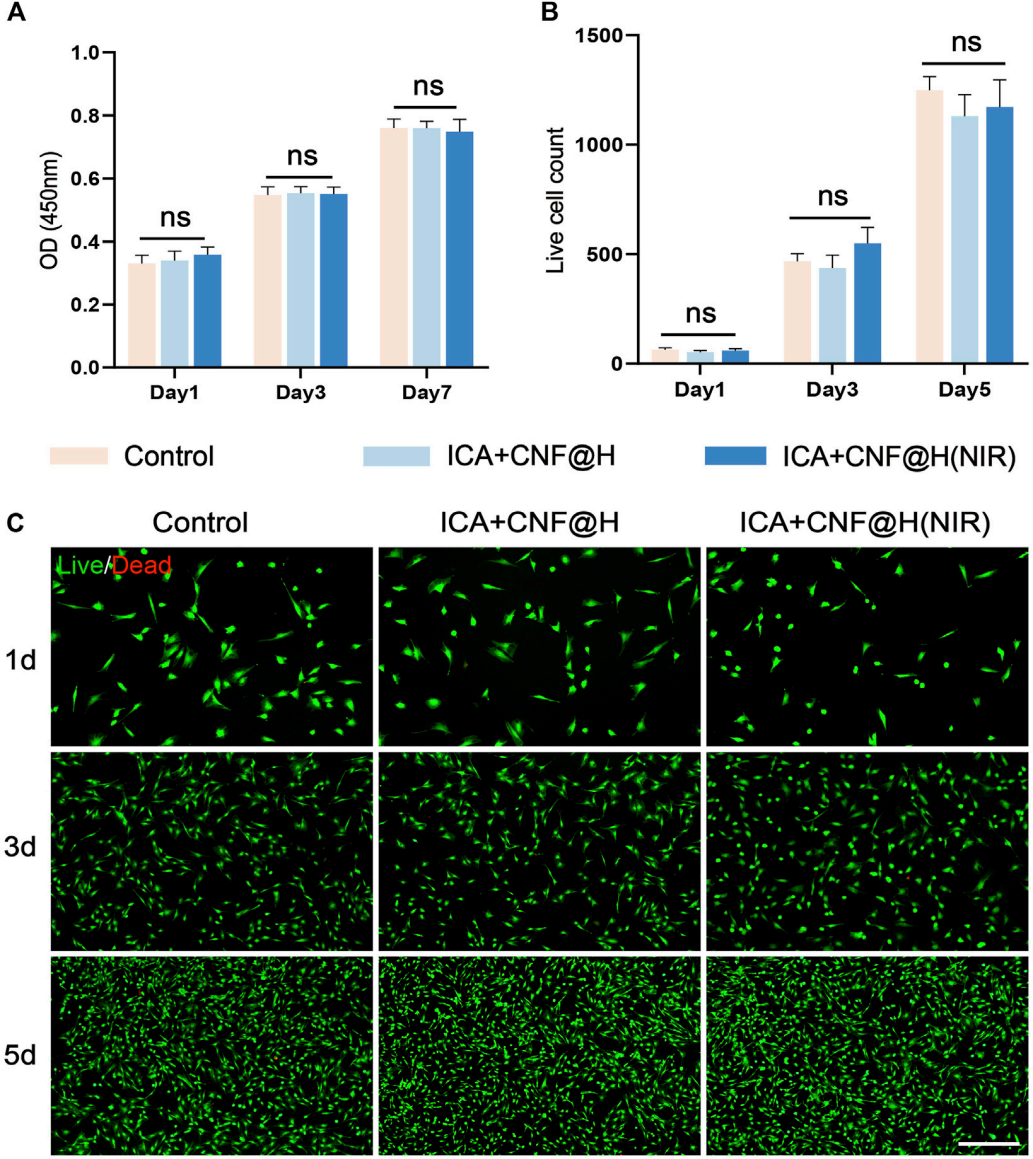
*In vitro* cell survival and cytotoxicity. **(A)** Results of CCK-8 in different groups. **(B)** Live/Dead fluorescent staining of counted cells. **(C)** Representative fluorescent pictures viewed through live/dead staining are revealed; bar = 100 μm *p < 0.05, **p < 0.01, ***p < 0.001.

Cytocompatibility is the basis for *in vivo* applications. To further investigate the bio-compatibility of composites, we examined the influence of composites by CCK-8 kits on the cell viability of BMSCs. The results of CCK-8 revealed that there was no obvious difference in proliferative activity of cells in the ICA + CNF@H(NIR) group as opposed to the control group at 1, 3 and 7 days (Figure 3A). The Calcein AM/PI Cytotoxicity Assay Kit was used to test the biosafety of ICA + CNF@(NIR), and the results from the live/dead cell staining showed (Figures 3B,C) that most of the cells in the different subgroups were viable at days 1, 3 and 5, and the cell density increased with time, with no statistically significant difference between the groups. The experiment shows that ICA + CNF@H (NIR) has NIR good biocompatibility.

### 3.4 Experimental study of composite materials on RAW264.7 cells

**FIGURE 4:**
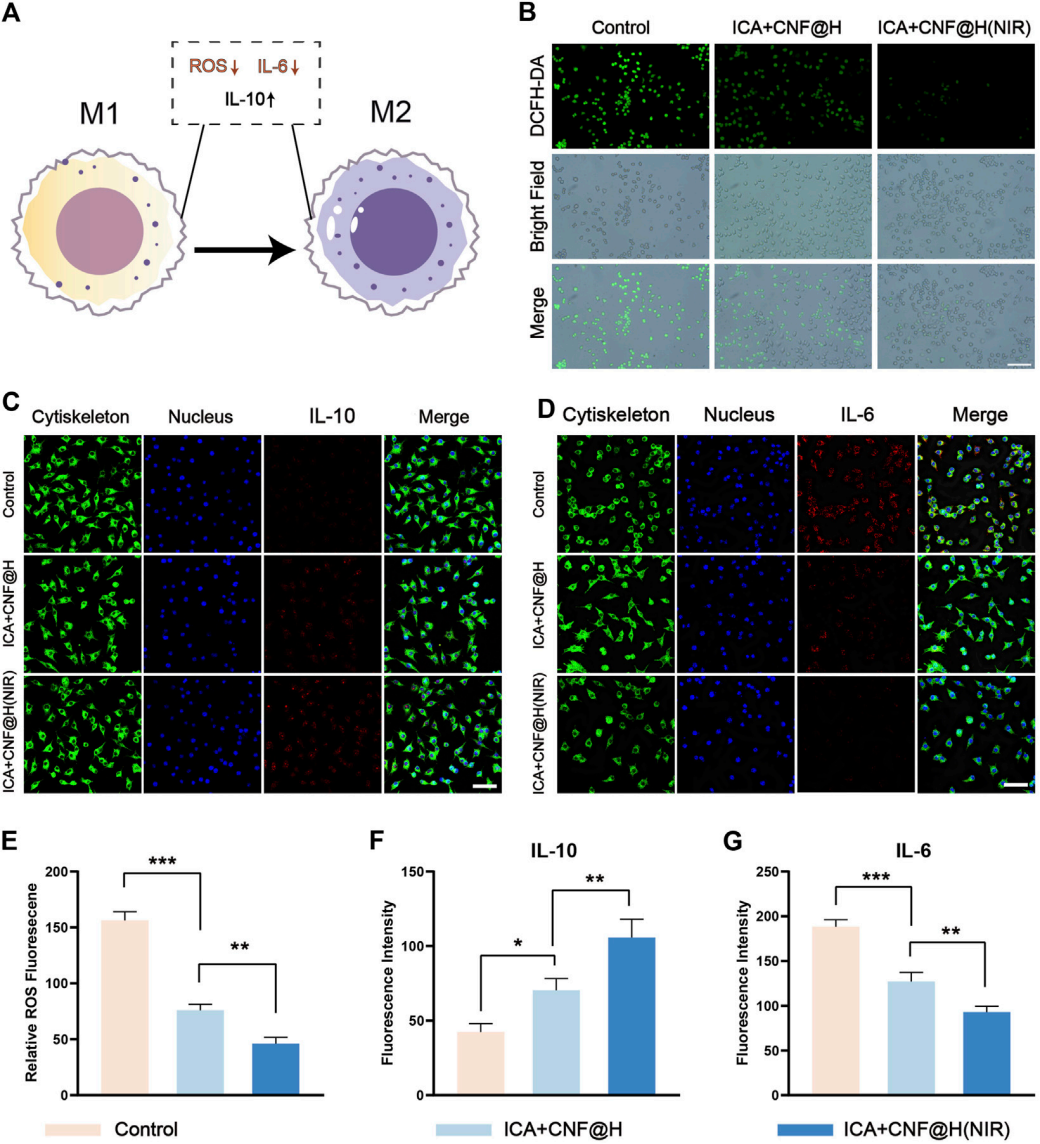
Experimental study of composite materials on RAW264.7 cells. **(A)** Macrophage polarization pattern map **(B)** Fluorescence image of ROS production by RAW264.7 cells; bar = 50 μm. **(C)**, **(D)** Immunofluorescence images of IL-10 and IL-6 in RAW264.7 cells; bar = 50 μm. **(E)** Semi-quantitative analysis of ROS in RAW264.7 cells. **(F)**, **(G)** Quantification of fluorescence intensity of IL-10 and IL-6. *p < 0.05, **p < 0.01, ***p < 0.001.

Periodontitis is defined as a chronic inflammatory disease of the periodontal supportive tissues primarily resulting from local factors. Macrophages have a key function in development of periodontitis (Zhuang et al., 2019; Yunna et al., 2020), M1 macrophages induce local inflammatory responses and release associated pro-inflammatory factors; M2 macrophages facilitate the repair of tissues and accelerate wound healing (Figure 4A). Macrophages M1 and M2 have a major function in inflammation and bone tissue healing, and induced M2 macrophages protect against bone loss. In vitro cell experiments (Figures 4C, D), with LPS stimulation of RAW264.7 as a control group, the ICA + CNF@H(NIR) group showed significantly lower expression of the pro-inflammatory factor IL-6 and significantly higher expression of the anti-inflammatory factor IL-10, promoting macrophage differentiation from M1 to M2. The pathogenesis of periodontitis is complex, but many investigations have demonstrated that increased local and systemic oxidative stress is directly related to the presence or severity of periodontal inflammation on site (Sczepanik et al., 2020). When reactive oxygen species are increased in cells, this leads to trophic stress and exacerbates periodontal tissue destruction (Wang et al., 2017; Sharifi-Rad et al., 2020). To test the antioxidant activity of ICA + CNF@H(NIR), we measured ROS levels in RAW264.7 cells after various grouping treatment with DCFH-DA fluorescent detectors and used LPS-stimulated RAW264.7 as a control group. The fluorescence imaging results showed (Figure 4B) that the green fluorescence was reduced to various degrees after different grouping treatments in comparison of the control group, but the green fluorescence of the composite was the weakest, indicating that the composite was better able to reduce reactive oxygen species, alleviate oxidative stress and mitigate periodontal tissue damage under the conditions of near-infrared light. The above results suggest that ICA + CNF@H (NIR) can reduce the inflammatory environment and help treat periodontitis relative to an inflammatory stimulated control group.

### 3.5 ICA + CNF@H(NIR) osteogenic differentiation *in vitro*

**FIGURE 5:**
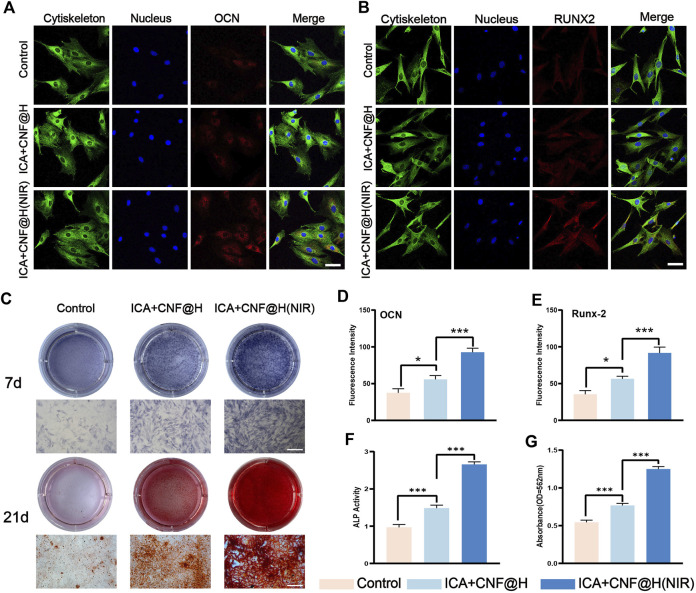
Experimental studies on BMSCs induced osteogenesis *in vitro*. **(A)**, **(B)** fluorescence images of OCN, RUNX2 of BMSCs at 7 days of induction; bar = 50 μm. **(C)** ALP was stained following 7 days of the induction, and alizarin red was stained following 21 days of induction; bar = 100 μm. **(D)**, **(E)** Quantification of fluorescence intensity of OCN and RUNX2. **(F)** Quantification of ALP activity 7 days after induction **(G)** Quantification of alizarin red staining of calcium nodules 21 days after induction. *p < 0.05, **p < 0.01, ***p < 0.001.

The prevention of alveolar resorption is a priority in the treatment of periodontitis. Osteoblasts represent the major functional cells in bone regeneration process and are primarily obtained from the derivation of bone marrow mesenchymal stem cells (Hu et al., 2022). For better periodontitis treatment results, it is important to promote the expression of cellular osteogenic factors in addition to controlling the inflammatory environment of the periodontium. Therefore, after demonstrating that the composite material has the ability to inhibit the expression of inflammatory factors, we further tested its osteogenic effect by treating BMSCs with control, ICA + CNF@H and ICA + CNF@H (NIR) groups for osteogenic induction. We evaluated how well the composites promoted osteogenic differentiation by measuring osteogenesis-related gene expression, ALP expression, and alizarin red calcified nodules in bone marrow mesenchymal cells. OCN is most rich non-collagenous protein in the bone and is mainly produced by osteoblasts. RUNX2 is a transcription element specialized for osteoblasts and plays a major part in the formation and reconstruction of bone tissue. OCN and RUNX2 play a key part in osteogenic OCN and RUNX2 play an indispensable role in osteogenic differentiation. Therefore, we examined on the expression of OCN and RUNX2 factors in BMSCs following 7 days of induction. The levels of OCN and RUNX2 expressed were markedly upregulated in the composite group in comparison to the control group (Figures 5A, B). Osteoblasts are capable of secreting ALP and synthesizing extracellular matrix such as type I collagen and osteocalcin to further mineralize and form bone tissue, and these characteristics were used as criteria to identify BMSCs differentiated into osteoblasts. Therefore, we performed a qualitative assay of the osteogenic differentiation capacity of the material by ALP staining. Only weak positive ALP staining was visible in the control group, while ALP staining was significantly stronger in the ICA + CNF@H and ICA + CNF@H(NIR) groups, and there was also a significant difference between ICA + CNF@H and ICA + CNF@H(NIR) (Figure 5C). Quantification of ALP activity after 7 days of induction in each component bone revealed that ALP expression activity was approximately 3-fold greater in the ICA + CNF@H(NIR) group than in control group (Figure 5F). The qualitative staining of posterior calcium nodules we examined with alizarin red staining, which forms a complex with calcium salts in a chelated manner to identify the calcium salt component of the tissue cells. Calcium salt changes are a marker of the proliferative differentiation of osteoblasts and the osteogenic potential of bone tissue. In each group, cells were stained with alizarin red and calcium nodules were quantified after 21 days of osteoblast induction (Figures 5C, G). The control group had more calcified deposits and formed twice as many calcified nodules compared to the ICA + CNF@H(NIR) group. Taken together, the results all indicate that ICA + CNF@H significantly promotes the osteogenic differentiation of BMSCs exposed to NIR.

**FIGURE 6:**
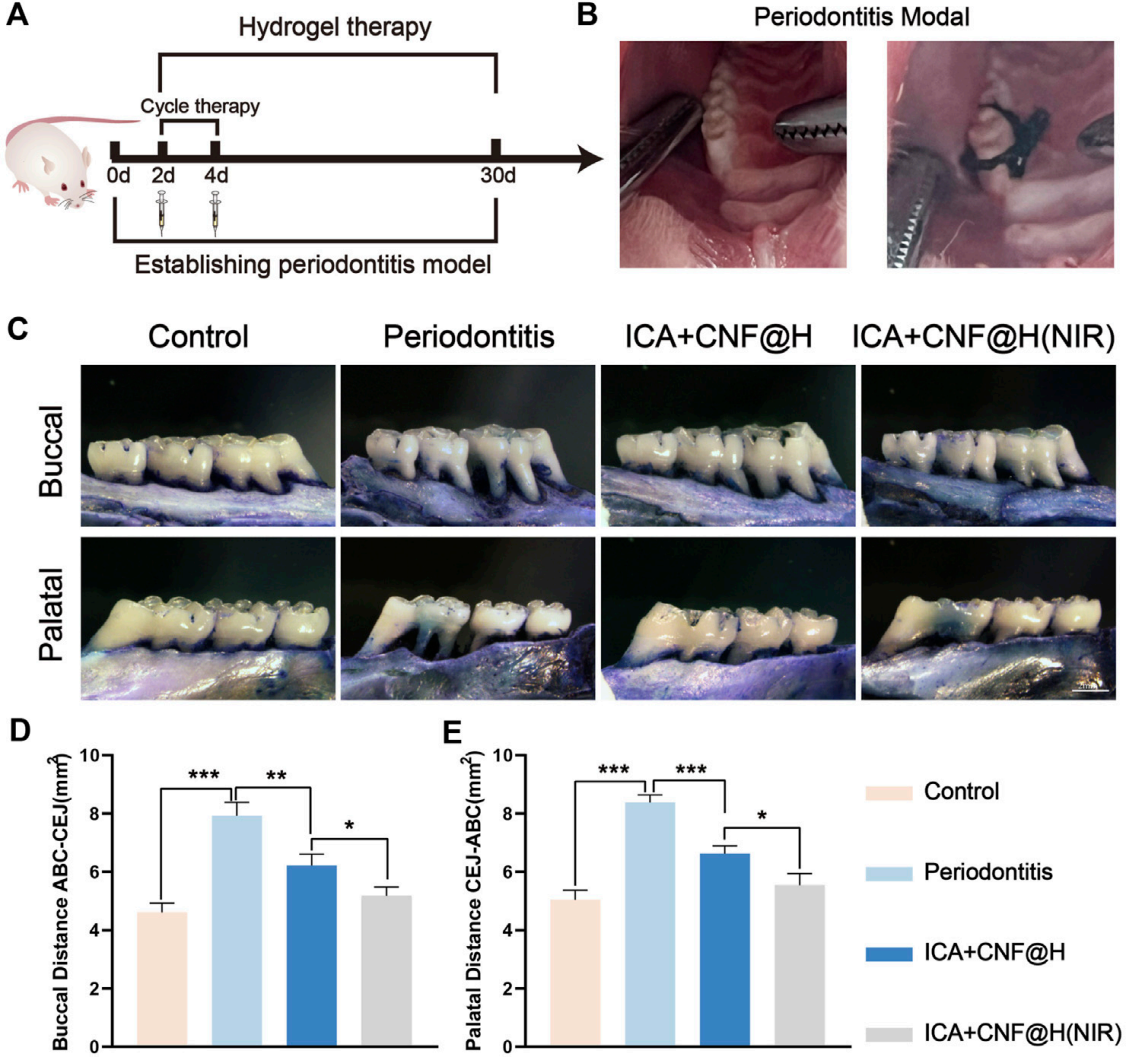
Efficacy of treatment of periodontitis. **(A)** Treatment periodogram and modeling images of rats. **(B)** Effectiveness of treatment of periodontitis in various subgroups; bars = 2 mm. **(C)** Quantitative ABC-CEJ of the buccal and palatal sides of the maxillary first molars. Quantification of the maxillary first molar buccal **(D)** and palatal **(E)** ABC-CEJ. *p < 0.05, **p < 0.01, ***p < 0.001.

In order to verify the role of ICA + CNF@H(NIR) in the treatment of periodontitis in an *in vitro* experiment, we conducted experiments in SD rats. In an *in vitro* animal model, an orthodontic ligature wire + filament ligation method was used to establish an experimental periodontitis model of the rat maxillary first molar (Figure 6B). Treatment was started the day after the periodontitis model was established. After randomly grouping and treating the rats in the blank, periodontitis, ICA + CNF@H and ICA + CNF@H (NIR) groups for 4 weeks, all rats were euthanized and maxillary tooth specimens were collected for subsequent experiments (Figure 6A). The treatment effect of ICA + CNF@H(NIR) was evaluated by microscopic observation and measurement of ABC-CEJ distances on the buccal and palatal sides of the maxillary first molars (Figure 6C). According to the symptoms of alveolar bone absorption, the height of alveolar bones was obviously reduced in periodontitis group in comparison with the controlling group, indicating the successful establishment of periodontitis model in rats. The ABC-CEJ distances of the buccal and palatal sides of each group were measured. The ABC-CEJ distances were shortened to different degrees after treatment in different groups, and the height of the alveolar bone was greatly increased in the ICA + CNF@H(NIR) group in comparison to the periodontitis group. The results suggest that the material has a better osteogenesis-promoting effect under NIR light irradiation.

**FIGURE 7:**
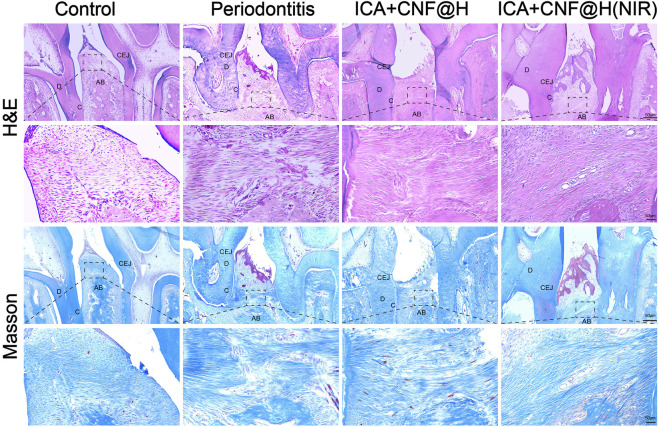
Periodontal tissue sections stained with H&E and Masson trichrome; bar=50μm. AB: alveolar bone; C: cementum; D: dentin; CEJ: cementoenamel junction.

Maxillary bone specimens were removed after different treatments, decalcified and sectioned for H&E and Masson trichrome to further observe histological morphology of the alveolar bone (Figure 7). As seen in the H&E staining, no alveolar bone resorption was seen in the blank group, the alveolar bone surface was smooth and the periodontal tissues were well arranged, compared to the blank group where the height of the alveolar bone was significantly reduced, the alveolar bone surface was not smooth and disordered connective tissue was visible in the local magnification of the periodontal tissues, indicating successful modeling. In the ICA + CNF@H(NIR) group, compared to the periodontitis group, the alveolar bone height was partially increased, the periodontal tissue damage was repaired, and the inflammatory condition of the periodontal tissue was partially relieved in comparison to control sample.

### 3.6 Discussion

Periodontitis has always been an oral disease that plagues patients. Periodontitis is a host-mediated inflammation that destroys the soft and hard tissues of the periodontium, leading to resorption of the periodontium and alveolar bone, and ultimately to tooth loss. The ultimate goal of periodontitis treatment is to control inflammation and achieve complete regeneration of periodontal tissues (Preshaw, 2004). ICA, a strong osteosynthesis agent, has been reported in previous studies to promote osteogenesis by stimulating osteogenic differentiation of bone marrow mesenchymal stem cells and is more effective and has fewer side effects than other flavonoid compounds. In recent years, carrier materials have been developed and new periodontal treatment materials will become a major research direction in periodontics. Topical application within periodontal pockets can effectively kill periodontopathogenic bacteria and eliminate the periodontal inflammatory environment. ICA has good biosafety and multiple pharmacological effects (He et al., 2020), can simultaneously kill bacteria and experiment with periodontal tissue regeneration, and combining these two modalities in the treatment of periodontitis is certainly a promising development. In this study, we prepared a novel multifunctional nanocomposite hydrogel in which ICA and CNF were loaded into an injectable hydrogel, which was irradiated with near infrared light to achieve better antibacterial, anti-inflammatory and osteogenic functions for the treatment of periodontitis. ICA itself also has good antibacterial properties CNF has strong NIR light absorption and meets the conditions of photothermal treatment. Under the irradiation of near infrared light, the light energy is converted into heat energy, causing irreparable damage to the bacteria. *In vitro* cell experiments have verified that the material has good biocompatibility and can promote the expression of osteogenic factors RUNX2 and OCN, demonstrating that the material can significantly promote the differentiation ability of BMSCs to osteoblasts and promote the maturation of osteoblasts and new bone formation function. In addition, it was shown that the material was effective in reducing the production of reactive oxygen species and the expression of inflammatory factors, and promoted the expression of anti-inflammatory factors to promote periodontal healing. In an *in vivo* experimental periodontitis model, the composite alleviated the destruction of alveolar bone. This study demonstrates the antibacterial, anti-inflammatory and osteogenic effects of the composite under NIR irradiation. The results of this study provide convincing evidence for the clinical usability of ICA + CNF@H(NIR) and offer new ideas for the treatment of periodontitis.

## Data Availability

The original contributions presented in the study are included in the article/Supplementary Material, further inquiries can be directed to the corresponding authors.
