# Supplementary material for: Photothermally responsive icariin and carbon nanofiber modified hydrogels for the treatment of periodontitis
Source: Front Bioeng Biotechnol. 2023 May 16;11:1207011. doi: 10.3389/fbioe.2023.1207011 (PMC10227505; doi:10.3389/fbioe.2023.1207011)
Supplement: Supplementary file 1 [file DataSheet1.docx]

**Supporting Information**


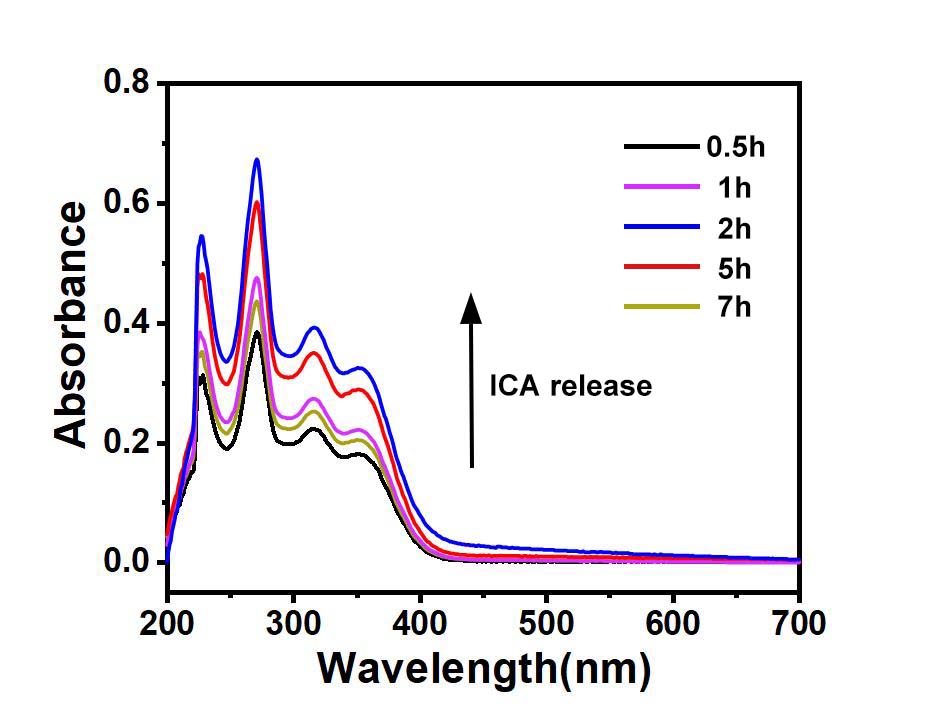


**Figure S1**. ICA release test of ICA+CNF@H.


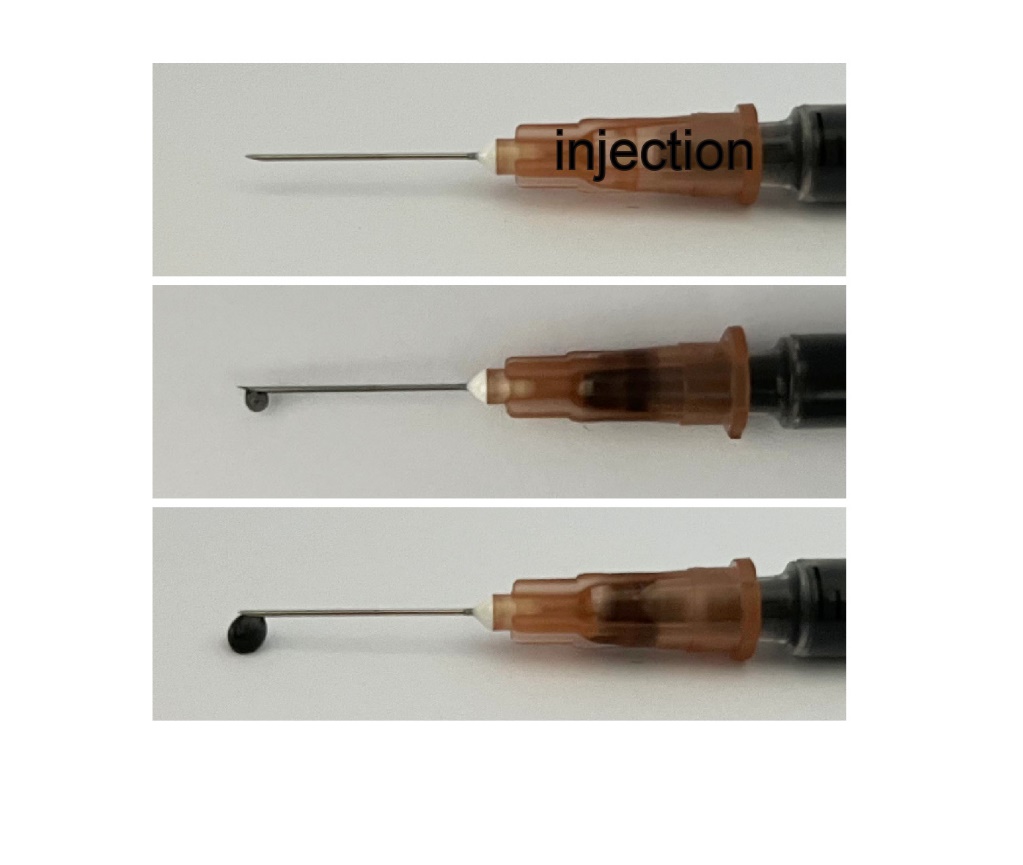


Figure S2. Testing of injectability of composite hydrogel system.
